# Associations Between Regional Factors and Cervical Cancer Screening Coverage in Tokyo

**DOI:** 10.31557/APJCP.2026.27.1.281

**Published:** 2026-01-21

**Authors:** Iori Harada, Yuri Ito, Kumiko Morita, Mizuki Kawahara, Shio Tsuda, Yu Kawabe, Rumi Tsukinoki

**Affiliations:** 1 *Department of Public Health Nursing, Insititute of Science, Tokyo, Japan.*; 2 *Department of Medical Statistics, Research & Development Center, Osaka Medical and Pharmaceutical University, Tokyo, Japan.*

**Keywords:** Cervical cancer, screening coverage, regional variation, health accessibility, human papillomavirus testing

## Abstract

**Objectives:**

Despite an increasing incidence of cervical cancer in Japan, screening coverage remains relatively low. This study aimed to elucidate regional variations in cervical cancer screening coverage in Tokyo, Japan, and to identify regional factors that influence screening coverage.

**Methods:**

This retrospective ecological study was conducted using data from 53 municipalities in Tokyo, sourced from the Tokyo Bureau of Public Health’s cancer screening statistics, population census, and official municipal government websites for the year 2018. We collected data on candidate regional factors in each municipality: including the proportion of working women, requirement for cancer screening appointments, and combination of cervical cytology and HPV testing, among others. The associations between screening coverage and these candidate factors were examined using a linear regression model with weighted least squares estimation.

**Results:**

The mean cervical cancer screening coverage in Tokyo was 18.6% (inter-municipal range: 8.1-85.8%). The following factors were significantly associated with cancer screening coverage: the number of medical institutions conducting screenings per 10,000 eligible women (β: 41.51, 95% CI: 23.66 to 59.36.0, P<0.001), proportion of working women (β: 1.03, 95% CI: 0.43 to 1.63, P<0.001), requirement for cancer screening appointments (reference: no requirement) (β: -5.56, 95% CI: -9.57 to -1.62, P=0.01), and inclusion of human papillomavirus testing (reference: no testing) (β: 12.89, 95% CI: 5.42 to 20.36, P<0.001).

**Conclusions:**

Cervical cancer screening coverage can be enhanced by improving accessibility to medical institutions that provide screenings, simplifying appointment procedures, and incorporating HPV testing. These findings provide valuable insights for designing effective public health policies and local screening strategies.

## Introduction

The incidence and mortality rates of cervical cancer have been increasing in Japan since the mid-1990s [1-3]. In 2020, Japan’s age-standardized incidence rate for cervical cancer was 15.2 per 100,000 women, which was substantially higher than that in most Asia-Pacific countries, including lower- and middle-income nations [4]. In Japan, despite the establishment of a national cervical cancer screening program in 1983 [5], screening coverage in Japan remains low when compared to the United States and Western Europe [2, 3, 6-8] and determinants of HPV infection have become increasingly prevalent[2].

Cervical cancer is highly preventable when cervical dysplasia is detected and treated before it becomes invasive. Screening programs commonly involve cytological examinations aimed at the early detection of precancerous changes that, if left untreated, may lead to cervical cancer. HPV testing has also been recommended as a primary screening method for cervical cancer for women aged 30 years and older [2]. As such, screenings represent one of the most useful methods for cancer prevention, ensuring high coverage is an important public health goal to support the reduction of cervical cancer incidence and cancer-related deaths. 

To facilitate participation in cancer screening programs, screening is offered at various settings across Japan. Cancer screenings conducted by municipalities can only be received at medical institutions or health centers designated by the local government. Local governments provide mass screenings, however, the target populations for these programs are often undefined. In addition, large companies and government organizations are responsible for providing screenings for their employees. However, participation in these screening programs is voluntary, and coverage rate is difficult to measure at the national level [9]. Recently, guidelines issued by Japan’s Ministry of Health, Labour and Welfare (MHLW) have recommend biennial cytological examinations of the cervix for women aged 20 years and older. The guideline also suggested guidelines on the inclusion of human papillomavirus (HPV) testing in primary screenings[10].At present, approaches to cervical cancer screening vary from region to region, which has led to geographical disparities in screening coverage [6]. 

Identifying regional-level factors that influence cervical cancer screening participation may help to inform the development of interventions to improve participation rates, thereby increasing overall coverage and reducing regional disparities [8]. However, studies have yet to explore the impact of such factors in Japan. This study aimed to elucidate regional variations in cervical cancer screening coverage (screening coverage) in Tokyo, Japan, and to identify regional factors that influence screening coverage.

## Materials and Methods

### Study design and setting

This study is a quantitative study. This retrospective ecological study evaluated cervical cancer screening rates in 51 municipalities (23 special wards, 28 cities or towns) in Tokyo, Japan, excluding nine towns located in remote island regions and two municipalities with missing data on regional factors (number of medical institutions conducting cervical cancer screening and necessity of screening appointments).

### Study outcome

The study outcome was screening coverage. We used data from the Tokyo Metropolitan Government’s Bureau of Public Health’s cancer screening statistics in 2018 [11] to determine each municipality’s screening coverage, number of eligible women, and number of women screened for cervical cancer. the screening coverage in this study did not include working women who underwent cancer screening provided by their large companies.

### Candidate regional factors

We analyzed the following candidate regional factors in each municipality: proportion of working women [12], number of medical institutions conducting cervical cancer screenings per 10,000 eligible women, cost of cervical cancer screening (free, 1–999 yen, or ≥1,000 yen), requirement for cancer screening appointments (yes or no), and combination of cervical cytology and HPV testing (yes or no). Each municipality’s residential population size and age (5-year age groups) of women were ascertained from the National Population Census [13]. We also collected data on the methods of cervical cancer screening from Tokyo’s cancer screening statistics [11]. Data on the number of medical institutions conducting cervical cancer screenings, cost of cervical cancer screening, and requirement for cervical cancer screening appointments were collected from each municipality’s official government website in 2023 after confirming that there were minimal differences from the data in 2018 [14].

### Statistical analysis

First, the regional variations in screening coverage were visualized on a map of Tokyo using MANDARA10, a geographic information analysis system software [15].

Next, we descriptively analyzed screening coverage and the candidate regional factors using mean values (inter-municipal range) for the continuous variables and number (percentage) for the categorical variables. The associations between screening coverage (percentage) and the candidate regional factors were examined using a linear regression model with weighted least squares. To adjust for variations in screening rates due to municipality size, the variance of screening rates was used as a weight, screening coverage the weighted variable was the square of the standard deviation of cancer screening coverage. The model was used to calculate the standardized regression coefficient (β) and 95% confidence interval (CI) of each candidate factor. P values below 0.05 were considered significant. All analyses were performed using SPSS version 24 (IBM Corp., Armonk, NY, USA).

## Results

**Figure 1:**
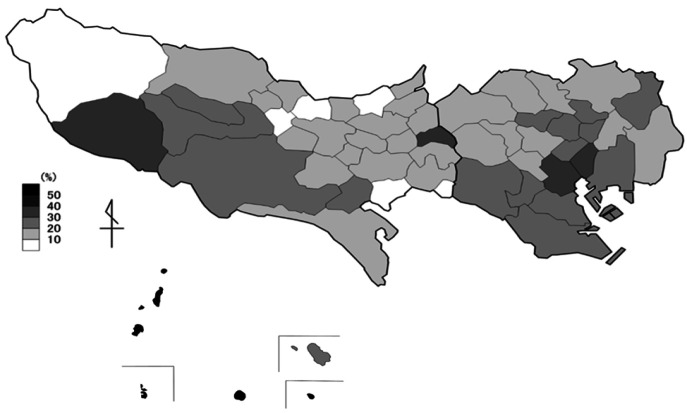
Regional Variations in Cervical Cancer Screening Coverage (%) in Tokyo in 2018.

The regional variations in screening coverage in Tokyo are shown in Figure 1. The overall mean screening coverage was 18.6%, with large variations across the municipalities (inter-municipal range: 8.1–85.8%).

Table 1 presents the descriptive statistics of screening coverage and the candidate regional factors. The mean proportion of working women across the municipalities was 38.9%. There were major differences in the number of medical institutions conducting screenings per 10,000 eligible women (inter-municipal range: 0.4–32.0%). Thirty-one municipalities (58.5%) offered free cancer screenings. Only three municipalities (5.7%) offered a combination of cervical cytology and HPV testing, two of which offered HPV-only testing for women aged 30–40 years. One municipality had adopted a method of performing cytological diagnosis and HPV testing (for women with findings of atypical squamous cells of undetermined significance or worse) using the same specimen. Twenty-one municipalities (39.6%) required cancer screening appointments for public health centers and hospitals.

Table 2 shows the associations between screening coverage and the candidate regional factors. The following regional factors were significantly associated with screening coverage: number of medical institutions conducting cervical cancer screenings per 10,000 eligible women (β: 41.51, 95% CI: 23.66 to 59.36.0, P<0.001), proportion of working women (β: 1.03, 95% CI: 0.43 to 1.63, P<0.001), requirement for cancer screening appointments (reference: no requirement) (β: -5.56, 95% CI: -9.57 to -1.62, P=0.01), and combination of cervical cytology and HPV testing (reference: no HPV testing) (β: 12.89, 95% CI: 5.42 to 20.36, P<0.001). No significant association was detected between screening cost and screening coverage.

## Discussion

This retrospective ecological study found that screening coverage was associated with accessibility to screenings and simple screening appointment procedures. This is consistent with the results of previous studies that have reported that accessibility to cervical cancer screenings has a substantial impact on coverage [16-22]. In Japan, cervical cancer screening is only conducted by obstetricians/gynecologists, screening institutions, or during group screenings at public health centers; and the screening system differs from municipality to municipality [5]. Our study demonstrated that the number of medical facilities that conduct cervical cancer screenings varied widely among the municipalities, which likely affects geographical accessibility and ease of making appointments. More complicated procedures for cancer screening appointments can also create a barrier to screening. We observed that almost 40% of the municipalities required appointments for cancer screening at public health centers and hospitals, such as through the use of screening vouchers. Our findings suggest that increasing the number of cervical cancers screening venues and simplifying appointment systems could help to facilitate screening participation in Tokyo.

Second, we observed that the proportion of working women residing in a municipality was associated with screening coverage. This supports previous findings that screening coverage tends to be higher among Japanese women aged 30–50 years [23], who represent the working generation that also coincides with the age in which cervical cancer is frequently diagnosed [24]. Another possible factor is that cervical cancer screening is often conducted simultaneously with antenatal health checkups in this age group. Additionally, the Tokyo Metropolitan Government and some workplaces may have implemented cervical cancer prevention strategies that target working-age women [11].

Furthermore, the present study also showed that screening coverage was associated with screening methods that incorporate HPV testing. Until 2023, Japanese guidelines only recommended HPV testing for those who required more precise cancer screening. The MHLW added HPV-only testing to screening guidelines in 2024 [10, 25]. Although it is difficult to manage both HPV-only testing and cervical cytology in cancer screening programs [10], the three municipalities in our study that offered a combination of cervical cytology and HPV testing may be actively focused on cervical cancer prevention, and have established cancer screening management systems that can monitor a variety of screening intervals.

Notably, our study found no clear association between cervical cancer screening costs and screening coverage. The cost of cervical cancer screening has been reported to be associated with screening participation [26]. However, we found that 58.5% of the municipalities offered free cervical cancer screenings and that the highest cost was still relatively low at only 2,120 yen. Therefore, it was unsurprising that the cost of screening was not a significant factor of screening coverage in Tokyo.

This study was strengthened by the use of data from both cancer screenings and official statistics in Tokyo’s municipalities. Moreover, this study is the first study to that elucidate regional variations in screening coverage in Japan. However, the findings should be interpreted with consideration to the following limitations. First, the study only focused on cervical cancer screenings within the municipalities of Tokyo—Japan’s largest metropolitan area—and may not be representative of Japan as a whole. Furthermore, our study did not include data from voluntary cervical cancer screenings conducted in the workplace. However, workplace screenings tend to have lower coverage than government-managed screenings. 

In conclusion, higher screening coverage in Tokyo’s municipalities was associated with ease of access to medical institutions conducting screenings, simplicity of screening appointment procedures, higher proportion of working women, and incorporation of HPV testing in screenings. These findings may help to inform the development of policies and programs to remove barriers to participation and increase screening coverage. Based on the results of this study, it is necessary to formulate cancer control measures that take into account regional diversity among local governments.

**Table 1:** Overview of Cervical Cancer Screening Coverage and the Candidate Regional Factors in Tokyo in 2018 (n=53)

	Mean	Inter-Municipal Range		
Cervical cancer screening coverage (%)	18.6	8.1	–	36.0
Proportion of working women (%)	38.9	31.9	–	44.8
Number of medical institutions conducting cervical cancer screenings per 10,000 eligible women†	3.2	0.4	–	32.0
Screening cost (yen)‡	338.1	0	–	2120
	n	%		
Screening cost (yen)‡				
0	31	58.5		
1–999	13	24.5		
≥1,000	9	17		
Requirement for cancer screening appointments for public health centers and hospitals†				
Yes	21	39.6		
Combination of cervical cytology and HPV testing				
Yes	3	5.7		

53 municipalities in Tokyo, Japan, excluding nine towns located in remote island regions; (please add before n=52)†n=52; ‡Screening cost refers to the mean cervical cancer screening costs for each municipality; HPV, human papillomavirus.

**Table 2:** Associations between Cervical Cancer Screening Coverage and the Candidate Regional Factors in Tokyo in 2018 (n=51)

	β	95% CI			P
Proportion of working women (%)	1.03	0.43	–	1.63	<0.01
Number of medical institutions conducting cervical cancer screenings per 10,000 eligible women	41.51	23.7	–	59.36	<0.01
Screening cost (yen) (Reference: 0 yen)					
1–999	-1.08	-6.13	–	3.98	0.67
≥1,000	-2.23	-7.32	–	2.86	0.38
Requirement for cancer screening appointments for public health centers and hospitals (Reference: No requirement)	-5.56	-9.57	–	-1.62	<0.01
Combination of cervical cytology and HPV testing (Reference: No HPV testing)	12.89	5.42	–	23.66	<0.01

CI, confidence interval; HPV, human papillomavirus.

## Author Contribution Statement

IH, YI, and RT conceived the study, planned the analytic approach, and conducted the study. IH collected the data and performed the data analysis. IH, YI, and RT created the tables and figure, interpreted the results, and drafted the manuscript. KM, YK, and ST helped to draft the manuscript. KM provided support for the statistical analysis. All authors revised the manuscript critically for important intellectual content and approved the final version.
